# Toxicity of Deepwater Horizon Source Oil and the Chemical Dispersant, Corexit® 9500, to Coral Larvae

**DOI:** 10.1371/journal.pone.0045574

**Published:** 2013-01-09

**Authors:** Gretchen Goodbody-Gringley, Dana L. Wetzel, Daniel Gillon, Erin Pulster, Allison Miller, Kim B. Ritchie

**Affiliations:** 1 Mote Marine Laboratory, Sarasota, Florida, United States of America; King Abdullah University of Science and Technology, Saudi Arabia

## Abstract

Acute catastrophic events can cause significant damage to marine environments in a short time period and may have devastating long-term impacts. In April 2010 the BP-operated Deepwater Horizon (DWH) offshore oil rig exploded, releasing an estimated 760 million liters of crude oil into the Gulf of Mexico. This study examines the potential effects of oil spill exposure on coral larvae of the Florida Keys. Larvae of the brooding coral, *Porites astreoides*, and the broadcast spawning coral, *Montastraea faveolata*, were exposed to multiple concentrations of BP Horizon source oil (crude, weathered and WAF), oil in combination with the dispersant Corexit® 9500 (CEWAF), and dispersant alone, and analyzed for behavior, settlement, and survival. Settlement and survival of *P. astreoides* and *M. faveolata* larvae decreased with increasing concentrations of WAF, CEWAF and Corexit® 9500, however the degree of the response varied by species and solution. *P. astreoides* larvae experienced decreased settlement and survival following exposure to 0.62 ppm source oil, while *M. faveolata* larvae were negatively impacted by 0.65, 1.34 and 1.5 ppm, suggesting that *P. astreoides* larvae may be more tolerant to WAF exposure than *M. faveolata* larvae. Exposure to medium and high concentrations of CEWAF (4.28/18.56 and 30.99/35.76 ppm) and dispersant Corexit® 9500 (50 and 100 ppm), significantly decreased larval settlement and survival for both species. Furthermore, exposure to Corexit® 9500 resulted in settlement failure and complete larval mortality after exposure to 50 and 100 ppm for *M. faveolata* and 100 ppm for *P. astreoides*. These results indicate that exposure of coral larvae to oil spill related contaminants, particularly the dispersant Corexit® 9500, has the potential to negatively impact coral settlement and survival, thereby affecting the resilience and recovery of coral reefs following exposure to oil and dispersants.

## Introduction

The Gulf of Mexico serves as a major source of crude oil for much of the Western hemisphere. It is estimated that over 1.5 million barrels of oil are extracted each day from offshore oil platforms in the Gulf [1], many of which are located within close proximity to the coastline. This intensive extraction and traffic of crude oil has, as seen with the Exxon Valdez and Deepwater Horizon (DWH) spills, the potential to result in large-scale environmental catastrophes with significant environmental impacts.

Remediation of oil spills often involves the application of dispersant chemicals, which can be mixtures of solvents and surface-active agents, along with other compounds. By reducing the interfacial tension between oil and water, dispersants enhance the breakup of an oil slick into small oil droplets that stabilize in the water column. While dispersants do not reduce the amount of oil entering the environment, they affect the fate, transport, and potential effects of an oil spill by altering the oil's physical properties [2].

The decision to use dispersant chemicals poses trade-offs for oil spill responders. While a dispersed surface oil slick is rendered less likely to reach the shore [3], treatment of major oil spills with dispersant chemicals has been shown to result in significant environmental degradation as a result of increased hydrocarbon dissolution and surfactant toxicity [4]. The effects of dispersed oil on the marine environment are also dependent on the degree of the dispersant's application as well as corresponding weather conditions [5]. While technological advancements have reduced the number of extraction- and transport-related incidents in recent years, the magnitude and potential impact of oil spills, such as the recent DWH spill in the Gulf of Mexico, affirms the fact that oil pollution constitutes a major threat to the marine environment [6], [7].

A major percentage of global offshore oil traffic occurs in close proximity to coral reef ecosystems [8]. The sensitivity of many coral species to environmental perturbation, as well as the current decline in reef cover worldwide has prompted a considerable amount of research into the effects of oil pollution on coral reef communities [7], [8], [9], [11]. Studies have shown that exposure of adult coral colonies to crude oil can result in a range of effects including inhibited growth rate, reduced reproductive activity, and tissue loss [10], [11]. Evidence also suggests that dispersed oil is significantly more toxic to corals than crude oil alone. Shafir et al. [7] found that in a survivorship assay of *Stylophora pistillata* and *Pocillopora damicornis* nubbins, concentrations of oil-dispersant mixtures above 25% caused 100% mortality to nubbins of both species, while none of the crude oil water-soluble fractions (WSF's) had any significant effect on coral survivorship.

While many field and laboratory experiments have noted significant damage to coral reef communities by oil contamination, the effects of oil exposure on coral reproduction and larval fitness and recruitment have received less attention. Loya and Rinkevich [11] noticed that exposure of *Stylophora pistillata* colonies to crude oil induced immediate mouth opening, followed by the premature release of underdeveloped larvae. Epstein et al. [4] found that exposure of *S. pistillata* planulae to increasing concentrations of dispersed oil resulted in reduced settlement and survivorship over the course of 96 hours. Similarly, Harrison [12] reported a reduction in metamorphosis of *Acropora tenuis* larvae when exposed to dispersed oil.

Coral larvae play a significant role in reef ecology. In scleractinian corals, planula larvae are the result of sexual reproduction, and have the ability to recruit to new substrate and contribute to genetic diversity [13]. Successful settlement of coral larvae involves the sampling of available substrate, followed by adherence and metamorphosis into a competent juvenile polyp [14]. Evidence suggests that larval settlement is strongly influenced by chemical cues, which are believed to stem from naturally occurring biofilms on marine substratum [8], [15], [16]. Consequently, surfactants alone and in the presence of oil could alter the physical and chemical properties of the ideal biofilm required for settlement or interrupt these sensitive chemical cues. Given the importance of successful larval recruitment in maintaining the reef environment, it is imperative to gain a fundamental understanding of toxicological effects on larval ecology.

Methods for conducting toxicity tests of chemical pollutants such as oil and dispersants have been developed and applied to teleost larvae and other organisms [17], [18]. The purpose of the present study was to evaluate effects of exposure to the water accommodated fractions (WAFs) of DWH oil (fresh and weathered), chemically enhanced water accommodated fractions (CEWAFs) of the oil, and Corexit® 9500 dispersant on planula larvae of the scleractinian corals *Porites astreoides* and *Montastraea faveolata* from the Florida Keys. Both species are common on reefs throughout the Caribbean, where *P. astreoides* is an early succession species contributing to reef recovery, and *M. faveolata* is an important reef building species. These species also differ in mode of reproduction; *P. astreoides* is a brooding coral that undergoes internal fertilization and releases semi-mature planula larvae from January to September [19], while *M. faveolata* is a broadcasting coral that spawns gametes in synchrony 1–2 times a year (Aug./Sept.) and requires external fertilization [20]. The longevity and extent of an oil spill in the water column can vary based on weather conditions [21] and microbial interactions [5], and may persist for several days, weeks or years [22]. This study examined the effects of exposure to both fresh and weathered DWH oil and dispersants using short-term assays (≤96-hr) to monitor larval settlement rates, survivorship, and behavioral responses. While the Deepwater Horizon spill did not occur in close proximity to the Florida Keys, it is plausible that oil pollution from similar events could eventually reach these coral reefs via offshore current movement [23], thereby affecting reef health.

## Results

### Settlement, Behavior, and Survival Assays (Weathered Oil)

#### Effects of weathered oil exposure on larval settlement and survival

**Figure 1:**
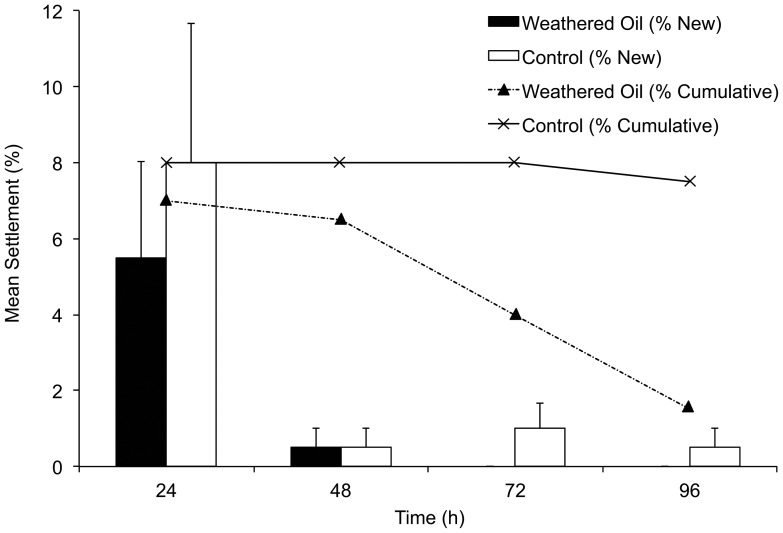
Settlement by larvae exposed to crude oil. Mean percent (% ± SE) new settlement by *P. astreoides* larvae exposed to Louisiana weathered crude oil (solid bars) and a seawater control (open bars) observed at each time point (24, 48, 72 and 96-hr). Mean percent (% ± SE) cumulative settlement by *P. astreoides* larvae after 24, 48, 72 and 96-hr exposure to Louisiana weathered crude oil (dashed line) and a seawater control (solid line).

The majority of *Porites astreoides* larval settlement observed in seawater control dishes and weathered oil applications occurred within the first 24-hrs; no significant difference was observed in settlement success between treatment types within that time period (p = 0.41; Student's *t*-test) (Fig. 1). Likewise, no significant differences were found in percent larval settlement between treatment and control dishes after 48 hours (p = 0.696; *F* = 0.154; repeated measures ANOVA). After 72-hr, no new settlement occurred by oil-exposed larvae, however, larvae in the control treatment continued to settle throughout the 96-hr observation period (Fig. 1).

Oil-exposed larvae experienced a greater rate of post-settlement mortality than control larvae (p<0.05; Student's *t*-test). At 72-hr, mean post-settlement survival of oil-exposed larvae had declined by 42% since the initial observation. At 96-hr, post-settlement survival had declined by almost 80% (p = 0.019; Student's *t*-test). While post-settlement survival in the control dishes remained relatively constant (survived) throughout the 96-hr observation period, it declined steadily in treatment dishes (Fig. 1).

#### Effects of weathered oil exposure on larval swimming behavior

Pairwise comparisons (Student's *t*-test) between the mean number of larvae located within each zone (0–6) and the number of larvae in the center of the dish did not differ significantly between oil treatment and control dishes (p>0.05), however larvae did show an overall preference for the outermost rim in all treatment dishes (p<0.001). On several occasions, larvae were observed contacting the oil mass perhaps as a possible substrate for settlement; neither settlement nor metamorphosis occurred amongst these larvae. Throughout the entire observation period no mortality was observed in any of the seawater control dishes or weathered oil exposure dishes.

#### Effects of weathered oil exposure on larval survivorship

Mortality of larvae in both acute (24-hr) and prolonged (72-hr) exposure vials was significantly higher than those in control vials (p = 0.0103; *F* = 4.039; repeated measures ANOVA). There was a 2% increase in mortality between the 24 and 48-hr exposures for the oil-exposed larvae, while no mortality was observed in the controls. After 72-hrs, natural mortality was observed in control larvae (4.5%). There was no significant difference between larval mortality associated with acute exposure (24-hr) and prolonged exposure (72-hr) (p = 0.297; *F* = 1.238; repeated measures ANOVA).

### Constant Exposure Settlement and Survival Assays

#### Effects of Water-Accommodated Fractions (WAF)

**Figure 2:**
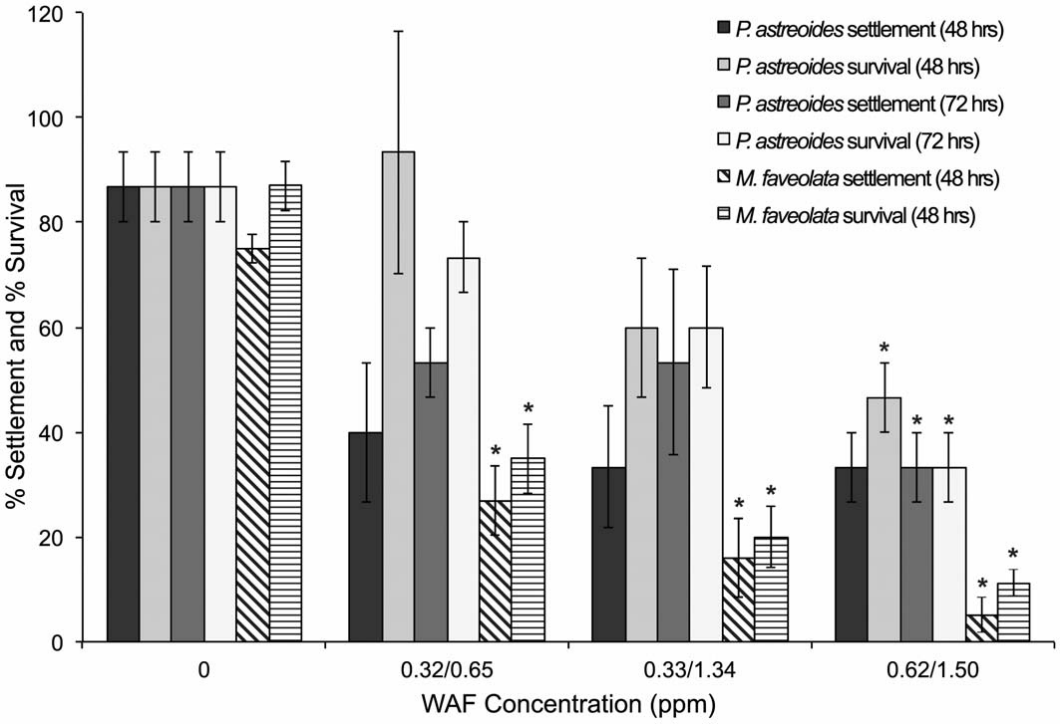
Settlement and survival of larvae exposed to WAF. Mean percent (% ± SE) settlement and survival of *P. astreoides* (solid bars) and *M. faveolata* (striped bars) larvae under constant exposure to DWH crude oil WAF (0, 0.32, 0.33, 0.62 ppm for *P. astreoides*; 0, 0.65, 1.34, 1.50 ppm for *M. faveolata*). Asterisks indicate comparisons that differ significantly from the control (ANOVA/Dunnett's; α = 0.05).

No correlation was found between WAF concentration and *P. astreoides* settlement after 48-hrs (linear regression; p = 0.061), however, a negative relationship was found among concentration and settlement after 72-hr exposure of *P. astreoides* larvae (linear regression; p = 0.003) and 48-hr exposure of *M. faveolata* larvae (linear regression; p<0.0001), where increasing WAF concentration was associated with decreased larval settlement. Settlement rates of *P. astreoides* larvae differed significantly among concentrations (ANOVA; p = 0.023; *F* = 3.301; Fig. 2). No significant effect on settlement occurred after 48-hr exposure; however, 72-hr exposure to high concentrations of WAF [0.62 ppm] resulted in significantly reduced larval settlement rates of 33% compared to 87% in control treatments (Dunnett's; α = 0.05; Table 1). Settlement of *M. faveolata* was significantly reduced by exposure to all three WAFs (ANOVA; p<0.0001; *F* = 31.92; Fig. 2), where 48-hr exposure resulted in 27% mean larval settlement at 0.65 ppm, 16% at 1.34 ppm, and 5% at 1.50 ppm compared to 75% mean settlement in the control treatment (Dunnett's; α = 0.05).

**Table 1:** Mean percent of settled, swimming and surviving larvae after 48 and 72-hr constant exposure for *P. astreoides* (#/5 ± S.E.) and 48-hr constant exposure and 96-hr spiked exposure for *M. faveolata* (#/25 ± S.E.; #/5 ± S.E.) to WAF, CEWAF and Corexit® 9500.

	Concentration	% Settled ± S.E.		% Swimming ± S.E.		% Surviving ± S.E.	
*P.astreoides*	(ppm)	48 hours	72 Hours	48 hours	72 Hours	48 hours	72 Hours
Control	0	87±7	87±7	0±0	0±0	87±7	87±7
	0.32	40±23	53±7	53±29	20±20	93±23	73±13
WAF	0.33	33±13	53±18	27±18	7±7	60±12	60±12
	0.62	33±7	33±7	13±7	0±0	47±7	33±7
	0.71	67±13	67±13	0±0	0±0	67±13	67±13
CEWAF	4.28	0±0	20±12	27±13	7±7	27±13	27±13
	30.99	0±0	7±7	33±33	0±0	33±33	7±7
	25	40±12	33±7	27±7	33±7	67±7	67±7
Corexit®	50	7±7	13±7	33±33	0±0	40±31	13±7
9500	100	0±0	0±0	0±0	0±0	0±0	0±0

	Concentration	% Settled	% Swimming	% Surviving	Spiked Conc.	% Survival
*M. faveolata*	(ppm)	± S.E.	± S.E.	± S.E.	(ppm)	± S.E.
Control	0	75±3	12±2	87±5	0	73±7
	0.65	27±7	8±5	35±7	0.49	33±7
WAF	1.34	16±8	4±2	20±6	0.51	27±7
	1.5	5±4	5±1	11±3	0.84	7±7
	14.73	4±2	0±0	4±2	0.86	20±12
CEWAF	18.56	0±0	0±0	0±0	30.06	7±7
	35.76	1±1	0±0	1±1	42.08	0±0
	25	5±4	4±2	9±1	500	20±12
Corexit®	50	0±0	0±0	0±0	1000	0±0
9500	100	0±0	0±0	0±0	1500	0±0

A negative relationship was found among larval survival and WAF concentration for *P. astreoides* (linear regression; p = 0.008, 48-hrs; p = 0.001, 72-hrs) and *M. faveolata* (linear regression; p<0.0001), where increasing WAF concentration was associated with decreased larval survival. Survival of *P. astreoides* larvae varied significantly among WAFs (ANOVA; p = 0.003; *F* = 5.40; Fig. 2). Larval survival was significantly reduced after 48 and 72-hr exposure to only the highest [0.62 ppm] WAF concentrations [47% survival at 48-hr, 33% at 72-hr] compared to the control [87%] (Dunnett's; α = 0.05; Table 1). An LC_50_ of 0.51 ppm (95% C.L.s = 0.27–35.19) was determined. Larval survival by *M. faveolata* significantly declined after 48-hr exposure to all three concentrations of WAF (ANOVA; p<0.0001; *F* = 34.507; Fig. 2), resulting in mean larval settlement of 35% at 0.65 ppm, 20% at 1.34 ppm, and 11% at 1.50 ppm compared to 87% in the control treatment (Dunnett's; α = 0.05); the LC_50_ was 0.50 ppm (95% C.L.s = 0.16–0.70).

#### Effects of Water-Accommodated Fractions Plus Corexit® 9500 (CEWAF)

**Figure 3:**
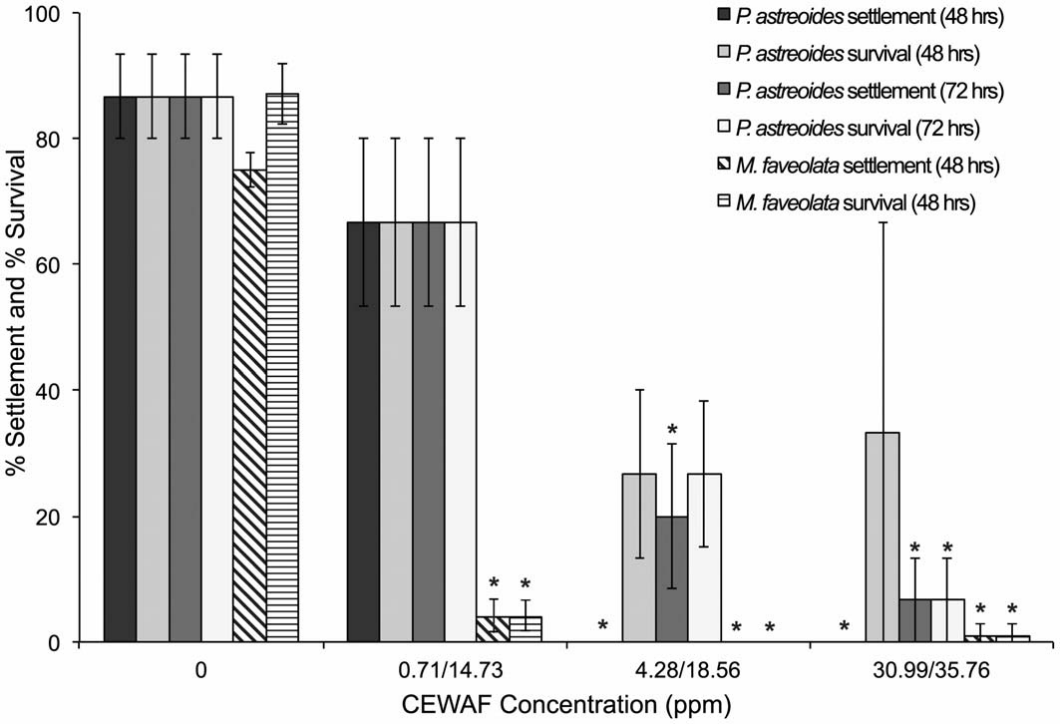
Settlement and survival of larvae exposed to CEWAF. Mean percent (% ± SE) settlement and survival of *P. astreoides* (solid bars) and *M. faveolata* (striped bars) larvae under constant exposure to chemically enhanced crude DWH oil CEWAF (0, 0.71, 4.28, 30.99 ppm for *P. astreoides*; 0, 14.73, 18.56, 35.76 ppm for *M. faveolata*). Asterisks indicate comparisons that differ significantly from the control (ANOVA/Dunnett's [*P. astreoides* settlement and survival/*M. faveolata* settlement]; Kruskal-Wallis/Dunn's [*M. faveolata* survival]; α = 0.05).

Linear regressions revealed a negative relationship among settlement and CEWAF concentration for *P. astreoides* (p = 0.001, 48-hrs; p<0.0001, 72-hrs) and *M. faveolata* (p = 0.040), where increasing CEWAF concentration was associated with decreased larval settlement. *P. astreoides* larval settlement differed significantly after exposure to various concentrations of CEWAFs (ANOVA; p<0.0001; *F* = 19.224; Fig. 3), where exposure to medium and high concentrations resulted in significant reductions after 48 and 72-hr exposure [0 and 20% mean settlement at 4.28 ppm, 0 and 7% at 30.99 ppm] compared to the seawater control [87%] (Dunnett's; α 0.05; Table 1). No effect was found on settlement, however, by the lowest concentrations of CEWAF [0.71 ppm], in which 67% mean settlement occurred after 48 and 72-hrs. Settlement of *M. faveolata* was significantly reduced by exposure to all three CEWAF concentrations (ANOVA; p<0.0001; *F* = 176.157; Fig. 3), where 4% mean settlement occurred at 14.73 ppm, 0% at 18.56 ppm, and 1% at 35.76 ppm compared to 75% in the control treatment (Dunnett's; α = 0.05).

No correlation was found between CEWAF concentration and *P. astreoides* survival after 48-hrs (linear regression; p = 0.068), however, a negative relationship was found among concentration and survival after 72-hr exposure (linear regression; p<0.0001). Survival of *P. astreoides* larvae differed significantly after exposure to various concentrations of CEWAF (ANOVA; p = 0.013; *F* = 3.792; Fig. 3). No significant effect on survival occurred after 48-hr exposure; however, 72-hr exposure to medium and high concentrations of CEWAF [4.28 ppm, 30.99 ppm] resulted in significantly reduced larval survival rates of 27% and 7% compared to 87% in control treatments (Dunnett's; α = 0.05; Table 1). An LC_50_ of 1.84 ppm (95% C.L.s = 0.26–4.72) was determined.

Spearman correlation analyses revealed a negative relationship among *M. faveolata* larval survival and CEWAF concentration (p = 0.007), where increasing concentration was associated with decreased larval survival. *M. faveolata* larval survival varied significantly among CEWAFs (Kruskal-Wallis; p = 0.037; *df* = 3; Fig. 3), where survival declined significantly after exposure to medium and high concentrations [18.56 ppm, 35.76 ppm] compared to the control treatment [0 ppm], resulting in mean survival of 0 and 1% compared to 87% respectively (Dunn's; α = 0.05; Table 1). While survival in low concentrations [14.73 ppm] did not significantly differ from the control, mean larval survival after exposure was only 4%. The LC_50_ for this exposure was 0.28 ppm (no C.L.s were generated).

#### Effects of Dispersant Corexit® 9500

**Figure 4:**
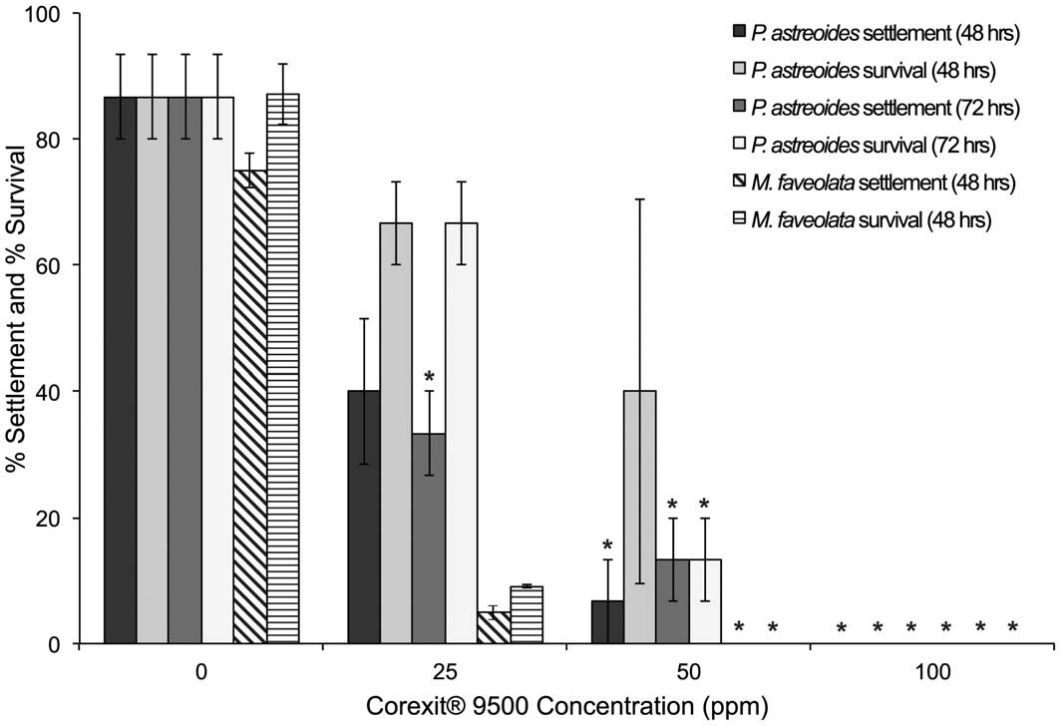
Settlement and survival of larvae exposed to Corexit® 9500. Mean percent (% ± SE) settlement and survival of *P. astreoides* (solid bars) and *M. faveolata* (striped bars) larvae under constant exposure to dispersant Corexit® 9500 [0, 25, 50, 100 ppm]. Asterisks indicate comparisons that differ significantly from the control (ANOVA/Dunnett's [*P. astreoides* settlement and survival]; Kruskal-Wallis/Dunn's [*M. faveolata* settlement and survival]; α = 0.05).

Linear regression and correlation analyses revealed a negative relationship among settlement and Corexit® 9500 concentration for *P. astreoides* (linear regression; p = 0.001, 48-hrs; p = 0.001, 72-hrs) and *M. faveolata* (Spearman rank; p<0.0001), where increasing Corexit® 9500 concentration was associated with decreased larval settlement. Significant differences were found among concentrations for *P. astreoides* larval settlement (ANOVA; p<0.0001; *F* = 29.143; Fig. 4), where overall settlement was significantly reduced after 48 and 72-hr exposure to all three concentrations of Corexit® 9500 [40 and 33% mean settlement at 25 ppm, 7 and 13% at 50 ppm, 0% at 100 ppm] compared to the control [87% at 0 ppm] (Dunnett's; α = 0.05; Table 1). Settlement rates of *M. faveolata* larvae also varied significantly among Corexit® 9500 concentrations (Kruskal-Wallis; p = 0.014; *df* = 3; Fig. 4), where significant reductions occurred in medium and high concentrations [50 ppm, 100 ppm] resulting in 0% mean settlement after 48-hr exposure (Dunn's; α = 0.05; Table 1). *M. faveolata* settlement was only 5% after exposure to low concentrations of Corexit® 9500 [25 ppm], however, this did not differ significantly from the control (Dunn's; α = 0.05).

A negative relationship was found between survival rate and Corexit® 9500 concentration for *P. astreoides* (linear regression; p<0.0001, 48-hrs; p<0.0001, 72-hrs) and *M. faveolata* (Spearman rank; p<0.0001), where increasing Corexit® 9500 concentration was associated with decreased larval survival. Survival of *P. astreoides* larvae differed significantly after exposure to various concentrations of Corexit® 9500 dispersant (ANOVA; p<0.0001; F = 9.385; Fig. 4), where exposure to high [100 ppm] concentrations of dispersant resulted in significant declines in survival at 48-hrs, while exposure to medium [50 ppm] and high concentrations [100 ppm] significantly reduced survival at 72-hrs. Exposure to the medium concentrations resulted in 13% mean survival; exposure to high concentrations resulted in complete mortality (0% survival). Mean *P. astreoides* larval survival was reduced from 87% in control treatments to 67% after exposure to the lowest concentration of Corexit® 9500 [25 ppm]; however, this difference was not significant (Dunnett's; α = 0.05). Results of the constant exposure test indicated a 72-hr LC_50_ value of 33.4 ppm (95% C.L.s = 20.11–43.73). Likewise, survival by *M. faveolata* larvae varied significantly between Corexit® 9500 concentrations (Kruskal-Wallis; p = 0.014; *df* = 3; Fig. 4), where survival significantly declined after exposure to medium and high concentrations [50 ppm, 100 ppm] compared to the control (Dunn's; α = 0.05), resulting in complete mortality (0% survival). While low concentrations [25 ppm] did not significantly affect survival, exposure resulted in mean larval survival of 9%, compared to 87% in control treatments. The 72-hr LC_50_ for this constant exposure was 19.7 ppm (no C.L.s generated).

### Spiked Exposure Survival Assays

**Figure 5:**
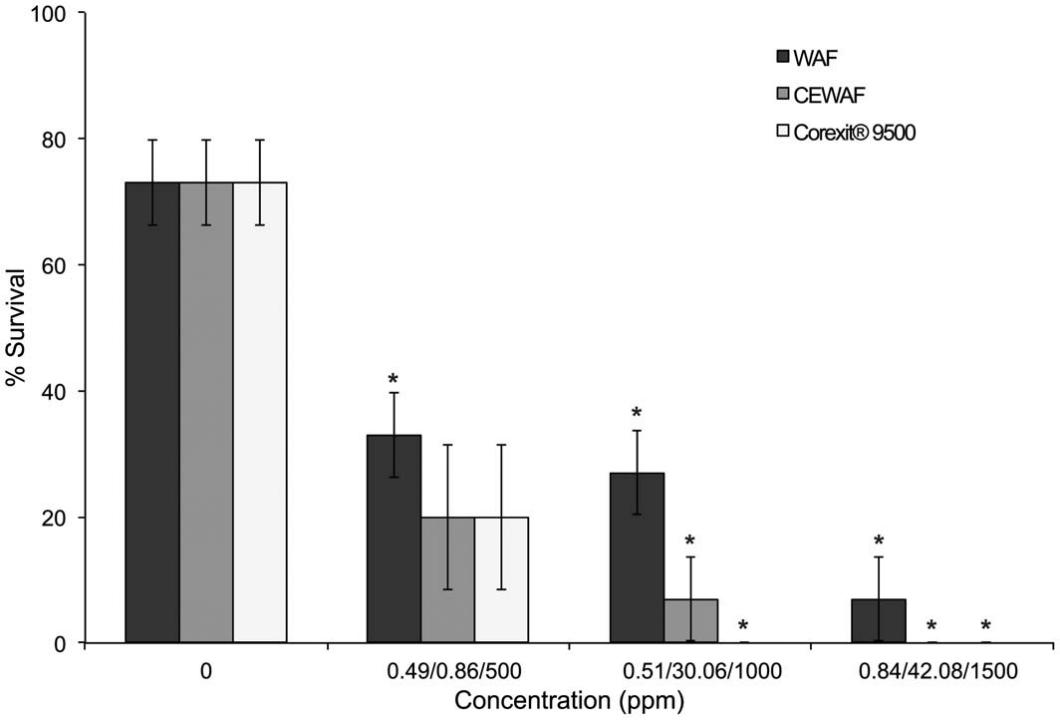
Survival of larvae in spiked exposure assays. Mean percent (% ± SE) survival of *M. faveolata* larvae after 96-hr spiked-declining exposure to WAF (0, 0.49, 0.51, 0.84 ppm), CEWAF (0, 0.86, 30.06, 42.08 ppb), and Corexit® 9500 (0, 500, 1000, 1500 ppm). Asterisks indicate comparisons that differed significantly from the control (ANOVA/Dunnett's [WAF]; Kruskal-Wallis/Dunn's [CEWAF and Corexit® 9500]; α = 0.05).

Correlation analyses of spiked exposure tests revealed a negative relationship among *M. faveolata* larval survival and concentration of WAF, CEWAF and Corexit® 9500 (linear regression, p<0.0001; Spearman rank, p = 0.001; Spearman rank, p<0.0001), where increasing concentrations were associated with decreased larval survival. Significant differences in *M. faveolata* survival were found among various WAF concentrations (ANOVA; p<0.0001; *F* = 17.583; Fig. 5), where survival was significantly reduced by exposure to all three concentrations of WAF [0.49 ppm, 0.51 ppm, 0.84 ppm] compared to the control treatment (Dunnett's; α = 0.05; Table 1) resulting in mean larval survival of 33%, 27% and 7% compared to 73% survival in the control, with a 96-hr LC_50_ of 0.45 ppm (no C.L.s were generated). Additionally, larval survival differed significantly among concentrations of CEWAF (Kruskal-Wallis; p = 0.037; *df* = 3; Fig. 5). Survival declined significantly after exposure to medium and high concentrations of CEWAF [30.06 ppm, 42.08 ppm] compared to the control (Dunn's; α = 0.05), resulting in 7% mean larval survival in medium concentrations and complete mortality (0% survival) in high concentrations. While *M. faveolata* survival was reduced from 73% in control treatments to 20% in the lowest concentration for spiked CEWAF, this difference was not significant. Results of the spiked exposure test indicated a 96-hr LC_50_ value of 0.12 ppm (no C.L.s were generated). Survival by *M. faveolata* larvae also varied significantly between Corexit® 9500 concentrations (Kruskal-Wallis; p = 0.014; *df* = 3; Fig. 5), where medium and high concentrations of Corexit® 9500 [1000 ppm, 1500 ppm] significantly reduced survival resulting in complete mortality (0% survival) in both concentrations. Although not significant (Dunn's; α = 0.05), exposure to low concentrations of Corexit® 9500 [500 ppm] resulted in a mean larval survival of 20%, compared to 73% in control treatments. The spiked exposure resulted in a 96-hr LC_50_ of 343.8 ppm (95% C.L.s = 146.88–759.37; linear interpolation).

## Discussion

### Effects of DWH crude oil, weathered oil and *Water-Accommodated Fractions (WAF)*

Exposure of *P. astreoides* planulae to fresh DWH oil resulted in mortality of experimental larvae within the first 24-hrs of treatment, while mortality in control planulae was not observed until 72-hrs (Fig. 1). Weathered DWH oil exposure also resulted in significant reductions in post-settlement survival after 48-hrs of observation. Moreover, constant and spiked exposure to WAF resulted in decreased larval settlement success and survival for *P. astreoides* and *M. faveolata* with increasing WAF concentration. *P. astreoides* larvae appear, however, to be more tolerant to WAF exposure than *M. faveolata* larvae as they were only significantly affected after 72-hr exposure to the highest concentrations of WAF [0.62 ppm] and not by lower concentrations [0.32 ppm, 0.33 ppm], whereas *M. faveolata* larvae were negatively affected by all three concentrations in both constant and spiked exposure scenarios [0.65 ppm, 1.34 ppm, 1.50 ppm; 0.49 ppm, 0.51 ppm, 0.84 ppm] (Table 1; Figs. 2 & 5). Chia [24] demonstrated that survival of pelagic larvae after oil-pollution may be related to size; that is, larger larvae are expected to survive longer. In general, planula larvae of brooding species tend to be larger than those of broadcasting species [25]. Tolerance for oil exposure may, therefore, be influenced by the larger size of *P. astreoides* larvae (1 mm) [19] compared to *M. faveolata* larvae (avg. egg size 320 µm) [20]. This differential tolerance based on size is likely related to variations in respirations rates and available lipid reserves, which are known to scale with size [26], [27], as well as the potential inability of planktotrophic larvae to feed and lecithotrophic larvae to photosynthesize in polluted waters.

As oil from a spill interacts with waves, microbes, oxygen, sunlight and other factors, volatile components in the oil gradually evaporate. This process is called ‘weathering’. Weathered oil, as a result of loss of its water-soluble elements, is more viscous than fresh crude, and is often dense enough to sink [28]. Results of settlement and survival assays for larvae exposed to weathered DWH oil as well as the constant and spiked exposure experiments presented here indicate that although weathered oil may not be toxic to coral larvae, there exists the potential for weathered oil to detrimentally impact marine organisms suspended in the water column in its proximity.

### Effects of *Water-Accommodated Fractions Plus* Corexit® 9500 (CEWAF)

Short and long-term exposure to medium and high concentrations of CEWAF [4.28 ppm, 30.99 ppm] resulted in significant reductions to both settlement and survival of *P. astreoides* larvae (Table 1; Fig. 3). Likewise, both the survival and settlement of *M. faveolata* larvae were negatively impacted by constant and spiked exposure to all three concentrations of CEWAF [14.73 ppm, 18.56 ppm, 35.76 ppm; 0.86 ppm, 30.06 ppm, 42.08 ppm] (Table 1; Fig. 3 & 5), where constant exposure to medium and high concentrations resulted in complete (0% survival) and near complete (1% survival) mortality. Moreover, near complete mortality (7% survival) occurred after spiked exposure of *M. faveolata* larvae to medium concentrations [30.06 ppm], and complete mortality (0% survival) occurred after spiked exposure to high concentrations of CEWAF [42.08 ppm]. These results indicate a negative relationship among larval settlement and survival with increasing CEWAF concentration and suggest that the application of dispersants to crude oil potentially increases the toxicity of oil exposure for coral larvae. The role of dispersants in mitigating oil spills is to break down the oil slick into stable droplets in the water column, thereby enhancing its rate of dissolution. The addition of dispersants, however, has the potential to compound the toxicological effects on marine organisms by increasing the surface area of oil-water interaction [29]. Likewise, dispersed oil is more evenly distributed throughout the water column and is, therefore, more likely to contact planktonic and benthic larvae, thereby increasing the potential negative impacts.

### Effects of dispersant Corexit® 9500

The results of the present study clearly indicate that dispersants are highly toxic to early life stages of coral. Settlement success and survival of *P. astreoides* and *M. faveolata* coral larvae were significantly reduced after both short and long term constant exposure, as well as spiked exposure, to increasing concentrations of Corexit® 9500 dispersant (Table 1; Fig. 4 & 5). Settlement of *P. astreoides* larvae, in particular, significantly decreased after exposure to all concentrations of dispersant [25 ppm, 50 ppm, 100 ppm], with no settlement occurring at the highest concentrations. Constant exposure to Corexit® 9500 also caused dramatic declines in larval survivorship, resulting in complete larval mortality (100%) after exposure to both medium and high concentrations [50 ppm, 100 ppm] and near complete mortality (9% survival) after exposure to low concentration [25 ppm] for *M. faveolata* larvae (Table 1; Figs. 4 & 5), as well as complete mortality under exposure to high concentrations [100 ppm] for *P. astreoides* larvae (Fig. 4). Similarly, *M. faveolata* larvae experienced complete mortality under spiked exposure to medium and high concentrations of Corexit® 9500 [1000 ppm, 1500 ppm], and only 20% survival under spiked exposure to low concentrations [500 ppm]. Complete mortality for both species after exposure to multiple concentrations of Corexit® 9500 in constant as well as spiked scenarios implies increased toxicity to larvae as compared to WAF and CEWAF. As previously noted, the differential results by species may be related to larval size, and thus larvae produced from broadcasting coral species may be more vulnerable to pollution than those from brooding species. Overall, these findings indicate that exposure of coral larvae to the dispersant Corexit® 9500 is toxic and will result in loss of coral recruitment.

### Conclusion

The explosion of the Deepwater Horizon oil rig in April 2010 resulted in the release of over 760 million liters of Louisiana crude oil into the Gulf of Mexico, thus constituting one of the greatest marine disasters in U.S. history [30]. Mitigation of the spill with dispersant chemicals was effective in reducing the magnitude of the offshore oil slick, however it is plausible that a significant portion of petroleum toxicants have been absorbed into the water column as a result. Much concern has arisen regarding the potential for oil pollution to reach coral reefs, particularly those in the Florida Keys that may be impacted by oil originating in the Gulf of Mexico and arriving via offshore currents.

Coral reefs worldwide have undergone drastic declines in the last several decades [31], [32]. This is particularly evident in the Caribbean, where coral cover has been reduced by roughly 80% since 1975 [33]. In the Florida Keys, coral reefs have been affected by anthropogenic and environmental impacts including pollution, overfishing, eutrophication, coastal development, disease, and climate change related bleaching among others. As a result, coral mortality in this region is unsustainably high [34] and substantially increased in 2010, following a cold-water event [35]. Such drivers have caused a dramatic shift in the Florida Keys reef ecosystem from a benthic community dominated by scleractinian corals to one overgrown with macroalgae [36]. With the advent of oil drilling off the coast of Cuba and our limited ability to be able to respond to it, coupled with the current fragile state of coral species in the Florida Keys, it is imperative that the potential impacts of oil pollution on Caribbean reef-building corals be understood at all life-history stages. This study found settlement and survival of *P. astreoides* and *M. faveolata* planulae decreased significantly following exposure to increased concentrations of DWH crude oil, weathered oil, WAF, CEWAF, and dispersant Corexit® 9500, with higher concentrations of CEWAF and Corexit® 9500 resulting in settlement failure and complete larval mortality. The demonstrated effects of pollution by DWH crude oil and the dispersant Corexit® 9500 on *P. astreoides* and *M. faveolata* planulae strongly suggest that the use of dispersants to mitigate oil spills in the vicinity of coral reefs should be avoided.

## Materials and Methods

Riser collected Deepwater Horizon (DWH) oil was obtained from British Petroleum (fresh) and weathered oil from oil masses obtained from Florida Wildlife Conservation from the spill site; Corexit® 9500 was obtained directly from the manufacturer (NALCO). Toxicity tests were performed to examine constant and spiked declining exposure to various concentrations of fresh DWH oil water-accommodated fractions (WAFs), chemically enhanced water-accommodated fractions (CEWAFs), and WAFs of Corexit® 9500. Effects studied were settlement and survivorship. Larvae were defined as settled upon observing successful metamorphosis from either free swimming or casually attached planula stage into a firmly attached, radially-symmetric disk shape form with pronounced flattening of the oral-aboral axis [14]. Larval mortality was defined as cessation of movement, followed by tissue degradation and dissolution. As overall guidance, the basic testing protocols of the American Society for Testing and Materials (ASTM) and the Organization for Economic Cooperation and Development (OECD) were followed as far as the routine steps in conducting a toxicity test. The test solution exposure concentrations are included in Table 2. The WAF and CEWAF actual concentrations were verified by gas chromatography-flame ionization detection (GC-FID) total hydrocarbon concentration (THC) analyses.

**Table 2:** Actual concentrations (ppm) of Corexit only and total petroleum hydrocarbons in WAF and CEWAF exposure solutions for experiments on *P. astreoides* and *M. faveolata* larval settlement and survival.

Solution		Actual Concentrations	
	*P. astreoides* Constant Exposure	*M. faveolata* Constant Exposure	*M. faveolata* Spiked Exposure
Corexit® 9500	25, 50, 100	25, 50, 100	500, 1000, 1500
WAF	0.32, 0.33, 0.62	0.65, 1.34, 1.50	0.49, 0.51, 0.84
CEWAF	0.71, 4.28, 30.99	14.73, 18.56, 35.76	0.86, 30.06, 42.08

### Larval collections

Two days prior to the new moon in both June and July of 2010, forty and twenty adult colonies of the mustard hill coral *P. astreoides*, approximately 30 cm×30 cm, were collected by hand from the mid-channel reef in the Lower Keys portion of the Florida Keys National Marine Sanctuary (24°33.6′N×81°30.1′W). Colonies were placed in outdoor flow-through seawater raceways at Mote Marine Laboratory Tropical Research Station in Summerland Key, FL. Each colony was isolated in a plastic container from which water flows into a collection apparatus, consisting of an 800 ml polypropylene beaker with 180 µm nylon mesh secured across the bottom. Larvae of *P. astreoides* float to the surface upon release and therefore overflow into the collecting beakers. Newly released larvae were washed from the collection cylinders into 1 L containers of 0.2 µm filtered seawater and counted. Larvae released from all forty colonies each night were pooled and then separated into glass scintillation vials consisting of 20 ml filtered seawater and maintained at room temperature (24°C) for experimentation.

Collection of gametes from adult *M. faveolata* colonies was done *in situ* using collection tents as described in Sharp et al. [37] in September of 2010 from Cheeca Rocks (24°54.4′N×81°37.6′W). Cross-fertilization was performed immediately after gamete collection at Long Key Marine Laboratory and embryos were maintained in filtered seawater for 5 days. Complete water changes were performed every 6 hours for the first 48 hours and subsequently every 8 hours. Developing larvae were then separated into glass scintillation vials consisting of 20 ml filtered seawater with 25 larvae per vial and maintained at room temperature (24°C) for experimentation.

### Experimental Procedure, Weathered Oil

Weathered oil masses were obtained from Florida Wildlife Conservation from the spill site. Acute-exposure tests were performed at room temperature (24°C) to examine the effects of weathered oil exposure on *P. astreoides* planulae. Effects studied were settlement and metamorphosis, swimming behavior, and survivorship (as defined above).

#### Settlement Assay

Oil effects on larval settlement and metamorphosis were examined in petri dishes filled with 10 mL of ambient seawater. Into each dish was placed a glass microscopy slide that was cured in the natural reef environment for three weeks prior to experiments to form biofilms. Approximately 35 mg of weathered DWH oil was placed in the center of each biofilm slide. Twenty *P. astreoides* planulae were then added to all dishes and subsequently observed daily for settlement, metamorphosis, attachment to dish/slide, and mortality over the course of 96-hrs.

#### Aversion Assay

Behavioral effects on *P. astreoides* planulae were examined by placing grids of 6 concentric circles oriented ∼0.9 cm apart under petri dishes filled with 15 mL of ambient seawater. Uniform amounts of weathered DWH oil, approximately 4 mg, were applied to the center of each treatment dish. Twenty *P. astreoides* larvae were subsequently added to each dish. Larval swimming behavior was assayed to investigate whether larvae would actively avoid the weathered oil, or exhibit any other behavioral abnormalities. Swimming behavior was scored photographically at the following intervals: 10 min, 30 min, 60 min, 6-hr, and subsequent 12-hr intervals for 3 days. Data were analyzed using a repeated measure analysis of variance (ANOVA). Significant comparisons were further analyzed using Student's *t* pairwise comparisons. Scoring was based on location in reference to the center of the dish using a numerical system (1 = center of dish, 6 = outer rim of dish).

#### Survivorship Assay

To assess exposure-related mortality among *P. astreoides* planulae, approximately 8.5 mg of weathered DWH oil were smeared onto the base of glass scintillation vials (replicated 10 times). Vials were then filled with 15 mL of ambient seawater. Twenty planulae were then added to each vial and left for 24-hr. Larvae and water from 5 of the 10 treatment vials were immediately transferred to sterile, oil-free vials to contrast acute exposure with prolonged exposure. Thus, 5 replicates were directly exposed to the smeared oil for 24 hours, while the other 5 replicates were directly exposed for 5 days. Water chemistry done on these vials indicated that there were no detectable levels of total petroleum hydrocarbons (TPHs) or polycyclic aromatic hydrocarbons (PAHs) present post experimentation. The same protocol was followed for control vials. Larval survivorship and metamorphosis were scored every 24 hours for 5 days and analyzed using a repeated measure analysis of variance (ANOVA). Significant comparisons were further analyzed using Student's *t* pairwise comparisons.

### Experimental Procedure, Crude Oil & Dispersant

#### Constant Exposure Procedure

Effects of fresh DWH oil on planula larvae were examined in 250 mL beakers each containing uniform-sized plaster tiles cured in the natural reef environment for 3 weeks to form biofilm on which the larvae could settle. After preparation, the appropriate WAF, CEWAF and dispersant test solutions were added to the containers, followed immediately by addition of the test organisms. Five *P. astreoides* planulae were added to each beaker, with 3 replications per treatment (n = 3). Larval settlement and mortality in each beaker was scored as the number of larvae settled or surviving relative to the original number of larvae (5) after 48 and 72-hrs. Data met all assumptions of normality and variance (Lilliefor's test; Levene's test; α = 0.05) and were compared among concentrations using linear regression analyses as well as Analysis of Variance (ANOVA). Significant differences were further analyzed using Dunnett's post-hoc comparisons.

The same experimental design was repeated in August 2010 using 25 *M. faveolata* planulae per beaker; settlement and mortality were scored as the number of larvae settled or surviving relative to the original number of larvae (25) after 48-hrs. Data from assays of settlement and survival under exposure to WAF, and settlement under exposure to CEWAF met all assumptions of normality and variance (Lilliefor's test; Levene's test; α = 0.05) and were compared among concentrations using linear regression analyses as well as Analysis of Variance (ANOVA). Significant comparisons were further analyzed using Dunnett's post-hoc comparisons. Data from assays of survival under exposure to CEWAF as well as settlement and survival under exposure to Corexit® 9500 did not meet the assumptions of normality and were analyzed using a Spearman rank correlation as well as a nonparametric Kruskal-Wallis test. Significant differences were further analyzed using Dunn's procedure for multiple pairwise comparisons. Unless indicated otherwise, all LC_50_ values were determined using maximum likelihood probit.

#### Flow-Through Toxicity Chambers System

A continuous-flow exposure system was developed by Singer et al. [38] and employed by the Chemical Response to Oil Spills Ecological Effects Research Forum (CROSERF) working group [17] [18] for assessing toxicity of oil and oil dispersants on marine organisms and was used for the spiked exposure experiments. This continuous-flow exposure system provides a useful approach for testing sensitive, early life stages of marine organisms exposed to constant concentrations of toxicants and provides a tool for testing dispersant toxicity under dynamic exposure regimes that are relevant to actual field conditions.

#### Flow-Through Spiked Exposure Procedure

Exposure chambers (270 ml) were filled with whole (undiluted) test solution, and animals were added to the chambers in random order at the appropriate density, the chambers were sealed and the test commenced with the dilution of all chambers with clean, aerated, filtered seawater at a rate of approximately 2 ml/min. Natural seawater was used for dilution at ambient salinity. Over the duration of the test, the test animals, and equipment were monitored for continuous operation within designated limits.

Three replicates of each of the three concentrations of fresh DWH oil WAF and CEWAF, along with a dispersant only WAF, and seawater control were performed with 5 *M. faveolata* larvae per replicate (Table 1). Larval survivorship was scored after 96 hours. Survival data after exposure to WAF met all assumptions of normality and variance (Lilliefor's test; Levene's test; α = 0.05) and were compared among concentrations using linear regression analyses as well as Analysis of Variance (ANOVA). Significant comparisons were further analyzed using Dunnett's post-hoc comparisons. Data from survival assays after exposure to CEWAF and Corexit® 9500 did not meet the assumptions of normality and were analyzed using Spearman rank correlation analyses as well as a nonparametric Kruskal-Wallis test. Significant differences were further analyzed using Dunn's procedure for multiple pairwise comparisons. Unless indicated otherwise, all LC_50_ values were determined using maximum likelihood probit.

### Chemical analyses

#### WAF and CE-WAF Solutions

Concentrations of the WAF, CEWAF, and dispersant for both spiked declining concentration and constant exposure were selected based in part upon available recommendations from the multi-agency Ecological Risk Assessment (ERA) workshops along with findings of the CROSERF working group [18], [39], [40]. These exposures of concern were based upon a dispersed oil spill trajectory model supplied by NOAA's Hazardous Materials Response Division (NOAA-HAZMAT) for spill research in Santa Barbara channel and Chesapeake Bay [18], [40]. Validation of concentrations of WAF and CEWAF test solutions for the exposure studies were confirmed analytically.

Petroleum hydrocarbons in the test solutions were extracted and analyzed using modified EPA Method 3510C and analyzed for PAHs as above, while THC were analyzed using EPA Method 8260. The results are reported based on integration of the FID signal over the entire hydrocarbon range from n-C9 to n-C42 and quantified using an internal standard. A performance-based quality-assurance and quality-control program, which includes the parallel analysis of procedural blanks, and matrix spikes were implemented to ensure data of the highest quality. The GC response was monitored every 10 to 12 samples with product check standards. Procedural blanks were checked to confirm they were clear of targeted analytes.
